# Emeritus-Professor Conwy Lloyd Morgan

**Published:** 1936

**Authors:** 


### Obituary

### EMERITUS-PROFESSOR CONWY LLOYD MORGAN, F.R.S., LL.D.

AT the ripe age of eighty-four Lloyd Morgan has passed away. He was for many years the distinguished and greatly-beloved Principal of University College, and when the College received its charter in 1909 converting it into Bristol University Lloyd Morgan was designated the first Vice-Chancellor. He relinquished this office almost immediately, and Sir Isambard Owen was appointed in his stead. His decision caused many regrets, but no doubt he realized that the duties of Vice-Chancellor would occupy his whole time and he preferred to continue his philosophical and psychological studies as the Professor of Philosophy in the University. His works on Comparative Psychology won for him a world-wide reputation, for he combined a singular knowledge of Natural History, Psychology and Philosophy. One of his last published books, entitled *Life, Mind and Spirit*, dealt comprehensively with the relationship between science and religion. His brilliant intellectual gifts were enhanced by a total absence of any intellectual arrogance. He was one of the most modest, kindly and dignified men who ever occupied a chair in the University College and University of Bristol. His discharge of his duties as Principal of University College was one of the most cogent factors in paving the way to the establishment of our University. It was his hand that guided the course of the College into its chartered existence, and his foresight was of incalculable value in drafting the first Ordinances and Regulations of the University.

Dr. F. J. Poynton, Consulting Physician to University College Hospital and Winford Orthopædic Hospital, writes of him as follows :—

"It is an honour I greatly appreciate that the Editor should have asked me to write a few lines about the late Professor Lloyd Morgan. It is all but fifty years since, as a student at Bristol University College, I studied Zoology under his supervision. Since that time I have never forgotten and have frequently mentioned the inspiration of his personality and his teaching. He was one of those who educated not so much by the facts that he taught as by the opening of one's mind to the real meaning of the subject he taught as a guide to all scientific thought. I have read that it was a misfortune that he had to spend so much of his fine brain on teaching elements to students, but I am not convinced this was the case, for the finest intelligence can but live a lifetime, and by handing on his methods of thought and approach to younger generations—an unusual gift in itself—his life's work remains in active use in many and different activities. He lives in his pupils, and furthermore he lives gratefully remembered by them. His quiet, gentle humour was a charm. On one occasion he told me to get a fresh herring for dissection, and to this day I see the twinkle in his eye when I arrived in the laboratory bearing the fish with all its important essentials for study removed by the fishmonger for edible purposes. A lasting memory is that tall, bearded figure with the features of a high intelligence, and the quiet, kindly manner of a master."

